# Pre-sternal Embryonic Dermoid Cyst: A Case Report

**DOI:** 10.7759/cureus.64030

**Published:** 2024-07-07

**Authors:** Mohammed Mhand, Abdelhakim Harouachi, Mahmoud Aberkane, Amal Bennani, Tariq Bouhout, Badr Serji

**Affiliations:** 1 Department of Surgical Oncology, Regional Oncology Center, Mohammed VI University Hospital Faculty of Medicine and Pharmacy, Oujda, MAR; 2 Department of Anatomopathology, Mohammed VI University Hospital Faculty of Medicine and Pharmacy, Oujda, MAR

**Keywords:** benign cyst, rare case, sternal, embryonic, dermoid cyst

## Abstract

Epidermoid and dermoid cysts are benign, usually slow-growing lesions classified as ectodermal inclusion cysts. These cysts form when epithelial remnants become trapped during the closure of the first and second branchial arch; however, a few cases are related to trauma or are iatrogenic. Diagnosis is made based on the cyst development history and imaging such as ultrasound. Surgical excision is the treatment of choice to avoid complications. We report a case report of a dermoid cyst in the pre-sternal region in a 17-year-old male patient.

## Introduction

Epidermoid and dermoid cysts are benign, usually slow-growing, lesions belonging to the family of ectodermal inclusion cysts [1]. Dermoid cysts contain skin appendages such as sebaceous and sweat glands, and keratin, while epidermoid cysts are composed of stratified squamous epithelium [2,3]. These cysts occur mainly in the midline of the skull and face, but their mid-sternal location is rare [4,5]. Diagnosis is made based on the history of cyst development and imaging, including ultrasound [6]. Surgical excision is the treatment of choice to avoid complications [7].

Herein, we report a rare case of dermoid cyst arising from the pre-sternal region, in a 17-year-old male patient. We discuss the clinical course and the challenges of diagnosis and treatment and present a brief literature review.

## Case presentation

**Figure 1:**
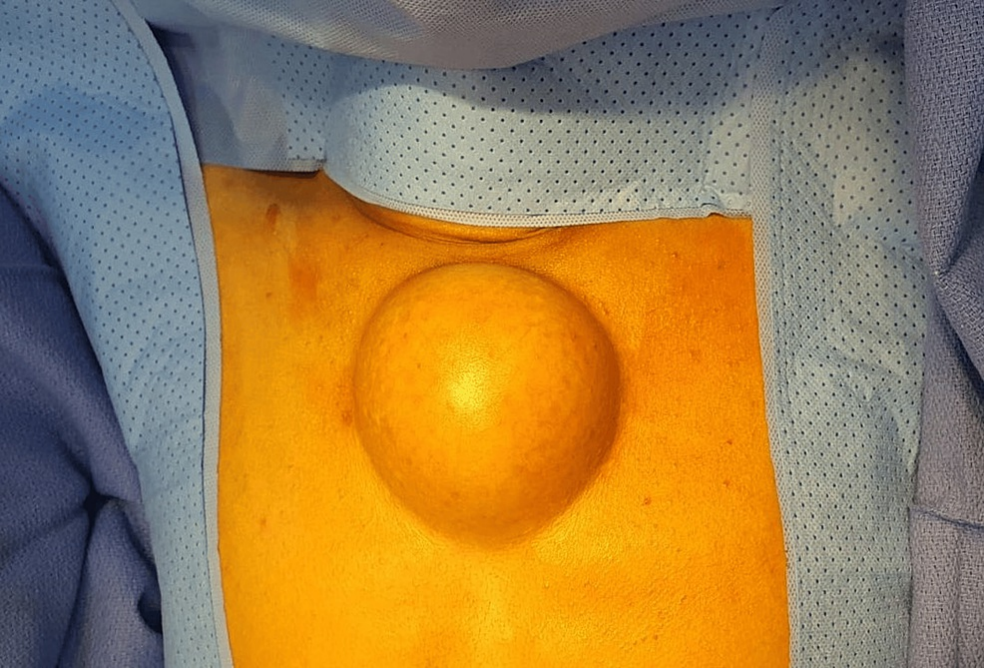
Pre-operative image of the mass

A 17-year-old male patient, without significant pathological history, presented with a subcutaneous mass in the pre-sternal region, which had gradually evolved in size since birth. The physical examination showed a well-defined oval mass on the anterior medial part of the thoracic cage, renitent, slightly painful on palpation, and mobile on the superficial and deep planes (Figure 1).

Notably absent were symptoms of fever or weight loss. There was no history of cancer in his family, and no use of drugs, alcohol, or tobacco. Laboratory tests, including liver biochemical tests, routine blood examination, hydatid serology, and serum tumor markers, were all within normal limits.

**Figure 2:**
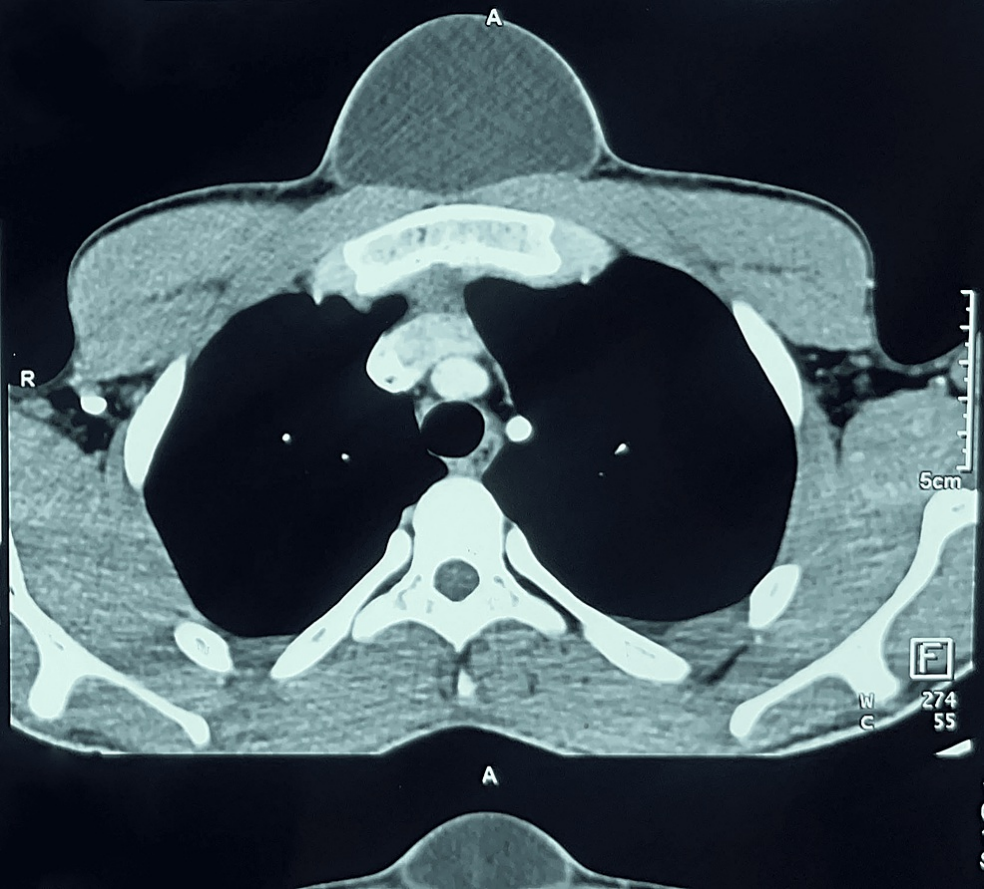
CT scan showing the pre-sternal mass (axial section)

**Figure 3:**
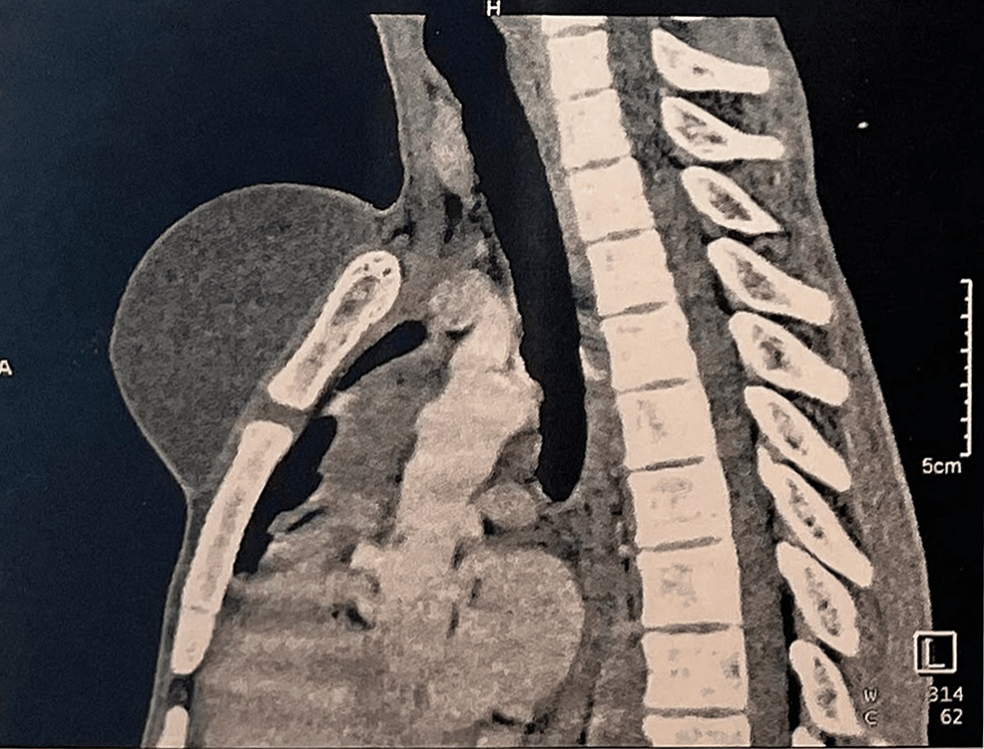
CT scan showing the pre-sternal mass (sagittal section)

Ultrasound and Doppler ultrasound found a cystic image with a mixed component of homogeneous, poorly defined, irregular, avascular echogenic areas (without color Doppler signal) bathed in clear liquid. Thoracic CT scan with injection of contrast showed a cystic mass in the soft subcutaneous parts of the anterior and median thoracic wall, measuring 68x50x94 mm, well defined, without parietal or bone infiltration (Figures 2, 3).

These findings were discussed in a multidisciplinary meeting among surgeons, radiologists, and oncologists. It was decided to surgically remove the mass. Thus, the mass was excised after obtaining the patient's consent was performed under general anesthesia. After an uneventful postoperative course, the patient was discharged four days after surgery

The anatomopathological results demonstrated a cystic formation bordered by a wall made of Malpighian epithelium surmounted by a thick layer of keratin lamellae, resting on a dermis sheltering pilosebaceous and sudoral appendages, and adipose tissue (Figure 3). The final diagnosis was a dermoid cyst. Presently, the patient is doing well, without recurrence after one year of follow-up.

**Figure 4:**
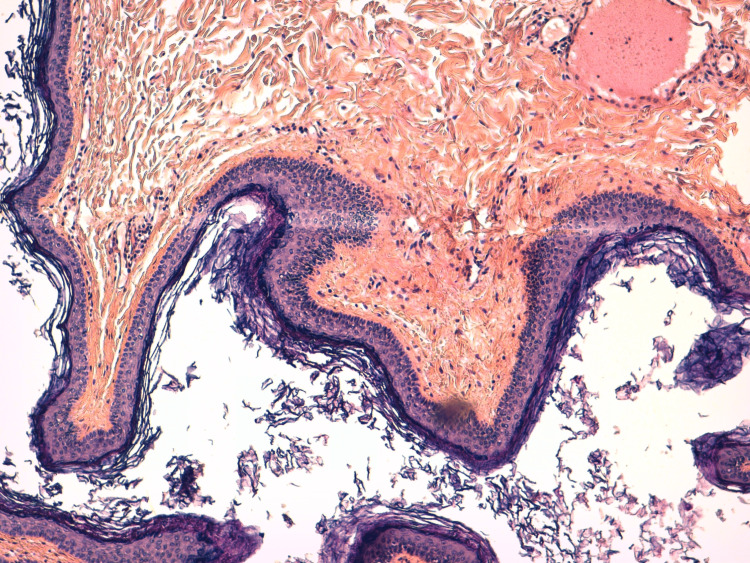
Histological section showing a fibrous cystic wall lined with keratinizing squamous epithelium and filled with keratin scales; hematoxylin-eosin-saffron (HES) stain x10

## Discussion

Dermoid cysts are benign entities of embryonic origin most found in individuals between 15 and 35 years of age. They rarely degenerate into a malignant form [8,9]. These cysts can be found anywhere in the body. They are externally axial fixed at one end or the other of the body (such as sacrococcyx or encephalon), paraxial most often gonadal, or internally axial (such as sella turcica or mediastinum). However, they are most found in the ovary (20%) and the testicles [10,11]. They affect men twice as often as women [9]. The etiopathogenesis of these cysts is not completely understood. During the closure of the first and second branchial arch, the inclusion of epithelial remnants leads to the formation of epidermoid cysts [12]; however, 10% of cases have been related to trauma or are iatrogenic [8].

The clinical symptomatology in the pre-sternal region is widely variable, depending on tumor size and proximity to vital structures [13]. Patients with dermoid cysts can remain asymptomatic for a long time. The main revealing symptoms are chest pain or the discovery of a mass [14]. Exploration by imaging such as ultrasound, computed tomography, or magnetic resonance imaging is crucial to visualize the cyst and its connections. On ultrasound, the epidermoid cyst is well-defined with a thick wall and echogenic debris. On CT scan it appears as a well-limited hypodense mass. On MRI, it appears as a hypointense lesion on the T1 sequence and hyperintense on T2 [15]. The gold standard of treatment is a complete surgical resection [14]. 

## Conclusions

When evaluating a pre-sternal cyst, dermoid cyst should be considered in the differential diagnosis. Dermoid cysts are rarely found in the pre-sternal region. Ultrasound is commonly used for initial evaluation and its features are as observed in our case.
